# Screening for Metabolic Dysfunction-Associated Steatotic Liver Disease in Individuals With Type 2 Diabetes Using Vibration-Controlled Transient Elastography and Serial Elastography Measurements to Monitor Response to Therapy: Methodological Details

**DOI:** 10.7759/cureus.101710

**Published:** 2026-01-16

**Authors:** Abhishek Singhai, Richa Meena, Rajnish Joshi, Sagar Khadanga, Ratinder Jhaj

**Affiliations:** 1 General Medicine, All India Institute of Medical Sciences, Bhopal, Bhopal, IND; 2 Pharmacology, All India Institute of Medical Sciences, Bhopal, Bhopal, IND

**Keywords:** fatty liver disease, fibroscan, masld, metabolic diseases, t2dm

## Abstract

Background: In type 2 diabetic mellitus (T2DM), metabolic dysfunction-associated steatotic liver disease (MASLD) is the most prevalent diffuse liver disease. Although most people with MASLD do not progress to the cirrhotic stage, patients with T2DM appear to advance more quickly and are at higher risk of steatohepatitis and liver-related mortality. The fibrosis degree is the strongest predictor of both liver-specific and all-cause mortality. Due to these factors, it is essential to identify people with advanced fibrosis or those who are more likely to develop it among the vast population of people with MASLD.

Methods: Vibration-controlled transient elastography (VCTE) will be used to determine the existence and severity of MASLD in people with T2DM and to examine its relationship to glycemic management and comorbidities associated with diabetes.

Results: Prevalence of MASLD among patients with T2DM will be measured using VCTE as an imaging modality, and the different stages of MASLD will be correlated with other diabetes related complications.

Conclusions: MASLD is a common liver disease in the diabetic population. Although Asians exhibit a considerably lower BMI and reduced obesity rates in contrast with other ethnic groups, they present an elevated prevalence of hypertension, diabetes, metabolic syndrome, and MASLD. This study is expected to estimate the prevalence of MASLD. A higher rate of MASLD is expected in diabetic individuals with poor glycemic control and who have other metabolic diseases.

## Introduction

India, home to 17.78% of the global population, carries a substantial share of the diabetes burden. India is the second most affected country in the world, with an estimated 101 million people living with type 2 diabetes mellitus (T2DM), or around 14% of the global prevalence [1]. An estimated 589 million persons between the ages of 20 and 79 had diabetes globally in 2024, with about 95% of those cases being T2DM. By 2050, the total population is anticipated to have increased by 46% to 853 million. About 89.8 million Indian individuals between the ages of 20 and 79 received a diabetes diagnosis in 2024, representing a prevalence of 10.5% and one in seven cases worldwide, according to the International Diabetes Federation's (IDF) Diabetes Atlas, 11th Edition (April 2025) [2]. Notably, 43% of these individuals, about 38.6 million, remained undiagnosed, underscoring the magnitude of the hidden disease burden in India [2]. The 2023 European Association for the Study of the Liver (EASL) Congress saw a significant change in disease terminology, with metabolic dysfunction-associated steatotic liver disease (MASLD) replacing nonalcoholic fatty liver disease (NAFLD). This reclassification was jointly endorsed by the European Association for the Study of the Liver (EASL), the Latin American Association for the Study of the Liver (ALEH), and the American Association for the Study of Liver Diseases (AASLD) [3]. MASLD promotes adverse cardio-metabolic outcomes through a range of pathophysiological processes, such as abnormal glucose metabolism, fatty acid metabolism, lipoprotein, adipokine, hypercoagulability, and atherosclerosis development. Patients with MASLD who also have T2DM, insulin resistance, hypertension, or other associated diseases are more likely to develop cardiovascular disease (CVD). At least half of all adults with type 2 diabetes develop steatohepatitis due to insulin resistance, regardless of whether they are obese or not [4]. When insulin resistance is severe, as in obesity and type 2 diabetes, the risk of developing steatohepatitis rises [5]. The substantial rise in the incidence of hepatocellular carcinoma (HCC) seen in diabetics is thought to be caused by steatohepatitis [6].

The primary objective of the study is to identify individuals with T2DM and evaluate them for MASLD using vibration-controlled transient elastography (VCTE). The secondary objectives of the study are: (i) To record parameters related to glycemic control and diabetes complications including cardiovascular disease, nephropathy, retinopathy, and neuropathy, (ii) To assess the correlation between stages of MASLD with glycemic status and diabetic complications, and (iii) To evaluate changes in steatosis and fibrosis scores following intensive glycemic control and lifestyle modifications.

## Materials and methods

This will be a hospital-based longitudinal study. Individuals above the age of 18 years who will be diagnosed with T2DM and who will be willing to undergo VCTE measurements will be included in the study. According to the 2019 American Diabetes Association guidelines, a patient is diagnosed with diabetes mellitus if their fasting plasma glucose levels are more than 126 mg/dl, their two-hour postprandial plasma glucose levels are more than 200 mg/dl, their HbA1C value is more than 6.5%, or their random plasma glucose is 200 mg/dl or higher and they exhibit the classic signs of hyperglycemia or hyperglycemic crisis and symptoms [7]. T2DM individuals are those who are receiving oral hypoglycemic drugs or not requiring insulin in the first six months of diagnosis. The following participants will be excluded from the study: (i) Patients with overt cirrhosis, (ii) Chronic hepatitis B with viral load >2000 IU/ml, (iii) Chronic hepatitis C with positive HCV RNA, (iv) Patients with active hepatitis (evident clinically), (v) Pregnant women, (vi) Patients using /have used hepatotoxic drugs in the last six months, (vii) Type 1 DM and secondary causes of DM, and (viii) Men consuming alcohol more than 210 gm/week of alcohol or women consuming alcohol more than 140 gm/week of alcohol. Patients having MASLD are advised lifestyle modifications, therapy for intensive glycemic control and control of comorbidities like dyslipidemia. All the parameters will be repeated at 6- and 12-month follow-up.

Sample size calculation

The formula for calculating sample size for prevalence studies (1.952*PQ/L2) is used to determine the sample size. Considering the prevalence of 65.3% of MASLD in type 2 diabetes mellitus [8], relative precision of 10% and 10% rejection rate, the calculated sample size is 588. The final sample size, assuming a 15% dropout rate, is 646. A purposive or convenience sampling technique is used.

Study protocol

In all study subjects, the following data will be collected: Data on demographic, socioeconomic, and behavioral characteristics, such as alcohol and tobacco usage, will be gathered through a standardized questionnaire. A complete medical history and family history of diabetes, duration of diabetes, medication use, and self-monitoring of blood glucose will also be obtained. Concurrently, a detailed clinical profile will be obtained for each subject by recording anthropometric data, vital parameters and biochemical markers, including HbA1c, lipid profiles, and renal function tests. The study will further screen for microvascular and macrovascular complications, such as retinopathy, nephropathy, and neuropathy, to facilitate a correlation analysis between the severity of liver involvement and diabetic complications. Following the baseline assessment, participants will undergo a structured intervention period focused on intensive glycemic control and standardized lifestyle modifications. At the conclusion of the intervention, follow-up VCTE measurements will be performed to quantitatively assess changes in hepatic steatosis and fibrosis, thereby evaluating the impact of metabolic optimization on liver health.

Fasting blood glucose, postprandial blood glucose, HbA1c, lipid profile, liver function test, serum creatinine, urine albumin creatinine ratio, ECG, monofilament test for neuropathy, fundus examination, and FibroScan parameters (controlled attenuation parameter, liver stiffness measurement) will also be measured. HbsAg and Anti-HCV will be checked only at baseline. The participants will be screened by VCTE (Fibroscan® expert 630 machines; Echosens, Paris, France) to diagnose MASLD. The velocity of a vibration wave-also referred to as a "shear wave"-generated on the skin is used to measure the hardness of the liver. The shear wave velocity is calculated based on the time required for a vibration wave to propagate to a specific depth within the liver. Because fibrotic tissue is stiffer than normal hepatic tissue, liver stiffness can be used to assess the degree of hepatic fibrosis. A trained technician positions the FibroScan probe in an intercostal space on the right side of the lower chest wall. The device delivers a series of painless mechanical pulses to the liver, and the measurements are recorded automatically. An overall liver stiffness value is generated and subsequently interpreted by a qualified physician to assess the likelihood of hepatic steatosis or fibrosis. FibroScan quantifies hepatic steatosis using the controlled attenuation parameter (CAP), expressed in decibels per meter (dB/m), and liver fibrosis using liver stiffness measurement (LSM) expressed in kilopascals (kPa). CAP values range from 100 to 400 dB/m and reflect the degree of hepatic fat accumulation. In healthy individuals, liver stiffness values typically range from 2 to 6 kPa, with a maximum measurable value of 75 kPa [9]. CAP scores measure liver steatosis, with cutoffs like <238 dB/m for normal, 238-260 dB/m (S1) for mild, 260-290 dB/m (S2) for moderate, and >290 dB/m (S3) for severe steatosis, indicating steatosis percentages from <11% to >67%; these scores help assess steatotic liver disease. LSM thresholds for fibrosis staging are categorized as follows: F0-F1 (no or minimal fibrosis) is defined by a measurement of less than 7.5 kPa; F2 (significant fibrosis) falls within the 7.6-9.9 kPa range; F3 (advanced bridging fibrosis) is indicated by 10.0-13.4 kPa; and F4 (cirrhosis) is confirmed at or above 13.5 kPa.

Data analysis

The collected data will be organized in Microsoft Excel and analyzed with IBM SPSS Statistics for Windows, Version 25 (Released 2017; IBM Corp., Armonk, New York, United States). Quantitative variables will be summarized as means and standard deviations with 95% confidence intervals, whilst qualitative variables will be provided as frequencies and percentages. Categorical variables will be reported as counts and percentages (%). Continuous variables are reported as mean ± SD for normally distributed data and median for skewed distributions. Logistic regression analysis will be used to find factors linked with the outcome variables.

Ethical approval has been obtained from the Institutional Human Ethics Committee (IHEC) for this study. Written informed consent will be obtained from all participants after explaining them study objectives and procedures.

## Results

Various modalities like serum markers, conventional imaging (ultrasound), and elastography (FibroScan) can be used for diagnosing MASLD in T2DM, each with distinct advantages and limitations. Serum biomarkers and non-invasive scores, such as the fatty liver index (FLI), hepatic steatosis index (HSI), and fibrosis scores like FIB-4 and APRI, use routine laboratory and clinical data to estimate steatosis and fibrosis; these are inexpensive and widely available but generally have moderate predictive accuracy, with AUROC values for steatosis markers around 0.65-0.68 and fibrosis scores showing variable performance (e.g., FIB-4 AUROC ~0.95 for advanced fibrosis) making them useful for initial risk stratification rather than definitive diagnosis [9]. Conventional ultrasound imaging detects hepatic steatosis by identifying increased echogenicity and is recommended as a first-line screening tool due to its broad accessibility and reasonable sensitivity, although its sensitivity decreases in patients with severe obesity and it cannot reliably stage fibrosis. In contrast, FibroScan quantitatively measures LSM and CAP, enabling more accurate assessment of both steatosis and fibrosis non-invasively. A comprehensive systematic review and meta-analysis of 20 studies (n≈6159 patients) showed that VCTE via FibroScan has good diagnostic accuracy for detecting both fibrosis and steatosis in MASLD across a range of settings; for example, for advanced fibrosis (≥F3), pooled sensitivity reached ~88.1% with AUC up to 0.84, and CAP accurately stratified steatosis stages S1-S3 with AUCs up to ~0.85. Probe type and patient BMI were significant covariates affecting performance [10]. In a clinicopathologic comparison, transient elastography (FibroScan) and magnetic resonance elastography (MRE) were both evaluated against biopsy-confirmed fibrosis. Although neither modality perfectly predicted high-grade fibrosis, FibroScan AUROC was ~0.75 for significant fibrosis, closely comparable to MRE (AUROC ~0.82), indicating solid non-invasive performance in clinical fibrosis staging [11].

## Discussion

The majority of MASLD patients do not exhibit any clinical symptoms in the early stage of the disease; later on, severe fibrosis may lead to liver failure. Thus, early diagnosis and management of MASLD are of vital importance. VCTE quantitatively measures liver stiffness, enabling a noninvasive clinical assessment of liver fibrosis and steatohepatitis. Most studies examining the prevalence of MASLD in T2DM in India have been conducted with small sample sizes or used USG as a modality of diagnosis. Our study with a large sample size and improved diagnostic method may result in more accurate results.

The prevalence of MASLD and advanced fibrosis among patients with T2D is strikingly high. Previous studies using VCTE in hospital settings have consistently reported high rates of steatosis and varying degrees of fibrosis in T2D cohorts, with MASLD present in approximately 55-65% of individuals and suspected advanced fibrosis in a notable minority (7-17%) depending on diagnostic thresholds and population characteristics [12,13]. Our results may align with recent systematic analyses confirming the diagnostic accuracy of VCTE for fibrosis staging in MASLD across diverse clinical settings. For significant fibrosis (≥F2), pooled diagnostic performance of VCTE showed acceptable area under the curve values and a strong negative predictive value, making it a suitable non-invasive initial screening tool in at-risk populations such as T2DM [14]. Similarly, meta-analyses have underlined the feasibility and point-of-care performance of VCTE in detecting both steatosis and fibrosis, reinforcing its role in early disease detection and management [10]. Longitudinal application of elastography provides insight into disease progression and therapeutic response. Large cohort studies suggest that serial VCTE measurements correlate with clinical outcomes: stable or reduced liver stiffness scores over time are associated with lower risk of liver-related events compared with persistently elevated values [15]. Although glycemic control appears to slow fibrosis progression in MASLD, regression of fibrosis and reduction in hard outcomes remain challenging, underscoring the need for liver-targeted therapies in addition to metabolic optimization [16]. Metabolic factors, including BMI and metabolic syndrome components, are strong determinants of steatosis severity and fibrosis risk in T2DM. Similar associations have been consistently reported in large multicenter cohorts and population studies, in which obesity and other cardiometabolic risk factors potentiate the MASLD burden [17]. Implementing structured screening programs in diabetes care facilitates earlier identification and management of fibrosis. 

Despite VCTE’s strengths, a subset of studies highlights limitations, including variability in cut-off values and occasional false positives requiring confirmatory assessment [18]. Limitations of our analysis include potential selection bias inherent in specialty referral populations and the lack of histologic confirmation. Moreover, while serial elastography offers meaningful insights into trends over time, optimal intervals and criteria for defining meaningful change remain to be standardized.

## Conclusions

MASLD is a common liver disease in the diabetic population. Although Asians exhibit a considerably lower BMI and reduced obesity rates in contrast with other ethnic groups, they present an elevated prevalence of hypertension, diabetes, metabolic syndrome, and MASLD. This study may show a similar or higher prevalence of MASLD in the local diabetic population. A higher rate of MASLD is expected in diabetic individuals with poor glycemic control and who have other metabolic diseases.

## Appendices

**Table 1:** Patient details

1	Patient Initials				
2	CR Number				
3	Age/Gender		4. Height:	5. Weight:	
6	Occupation:				
7	Contact:				
8	Address:				
	Treatment History				
9	Diagnosis:	10. Family History			11. Date:
	PERSONAL HISTORY				
12	Tobacco: Yes/No				
13	Smoking: Yes/No				
14	Alcohol: Yes/No, if Yes: type, quantity, frequency and duration of alcohol intake				
15	Sleep:				
16	Appetite:				
17	Bowel and Bladder habits:				
18	Comorbid Conditions: History of diabetes mellitus/hypertension/coronary artery disease/cerebrovascular accident/ chronic hepatitis B/chronic hepatitis C				
19	Hospitalization History				
20	Medication History				
21	Examination				
	General exam: Height: Weight: BMI: Waist: Waist-to-Hip Ratio:				
22	Pulse:				
23	BP:				
	Systemic exam				
24	CNS:				
25	CVS:				
26	Respiratory:				
27	Endocrine:				
28	GIT & Liver				
29	Renal:				
30	Musculoskeletal:				
31	Others:				

**Table 2:** Section B CAP:  controlled attenuation parameter; LSM: liver stiffness measurement

	Baseline	Month 6	Month 12
Height			
Weight			
BMI			
Waist			
Waist-to-hip ratio			
Fasting blood glucose			
Postprandial blood glucose			
HbA1c			
Liver Function Test			
Lipid profile			
HBsAg			
Anti HCV			
Serum creatinine			
Urine albumin creatinine ratio			
Fundus			
ECG			
Monofilament test			
FibroScan results CAP Score/LSM Score			
